# Agent-based model for Johne’s disease dynamics in a dairy herd

**DOI:** 10.1186/s13567-015-0195-y

**Published:** 2015-06-19

**Authors:** Jessica Robins, Sarah Bogen, Auldon Francis, Annet Westhoek, Andrew Kanarek, Suzanne Lenhart, Shigetoshi Eda

**Affiliations:** National Institute for Mathematical and Biological Synthesis, University of Tennessee Knoxville, 1122 Volunteer Blvd., Suite 106, Knoxville, TN 37996-3410 USA; University of Tennessee Institute of Agriculture, 2431 Joe Johnson Drive, 274 Ellington Plant Science Bldg, Knoxville, TN 37996-4563 USA; Capital University, 1 College Ave Bexley, OH USA; Wageningen University, 6708 PB Wageningen, The Netherlands

## Abstract

Johne’s disease is an infectious gastrointestinal disease in ruminants caused by *Mycobacterium avium* subsp. *paratuberculosis* that causes diarrhea, emaciation, decreased milk production and eventually death. The disease is transmitted in utero and via milk and colostrums to calves, and fecal-orally to all age classes. Financial losses due to the disease are estimated to be over $200 million in the US dairy industry. The goal of this study was to evaluate the cost effectiveness of control measures based on diagnosis with a sensitive ELISA, EVELISA. An agent-based, discrete time model was developed to simulate Johne’s disease dynamics in a US dairy herd. Spatial aspects of disease transmission were taken into account by using six spatial compartments. The effects on disease prevalence were studied with and without transmission routes included in the model. Further, using the model, cost effectiveness of ELISA-based Johne’s disease control was evaluated. Using the parameters we collected and assumed, our model showed the initial prevalence of Johne’s disease (33.1 ± 0.2%) in the farm increased to 87.7 ± 1.7% in a 10 year-simulation. When ELISA-based control measures were included in the simulation, the increase in prevalence was significantly slowed down, especially when EVELISA was used. However, the level of the prevalence was still higher than the initial level after 10 year simulation even with the ELISA-based diagnostic intervention. The prevalence was further reduced when quarterly ELISA testing was included. The cost analysis showed that the quarterly ELISA and EVELISA testing could bring $44.8 and $51.5/animal/year more revenues, respectively, to a dairy farm.

**Electronic supplementary material:**

The online version of this article (doi:10.1186/s13567-015-0195-y) contains supplementary material, which is available to authorized users.

## Introduction

Johne’s disease (JD) is an intestinal infection caused by *Mycobacterium avium* subsp. *paratuberculosis* (MAP) [1,2]. The disease infects wild [3] and domestic ruminants, including the dairy cow [1,2]. Symptoms include diarrhea, weight loss, decreased productivity and eventually death [1,2]. Johne’s disease has spread worldwide [1]. Of the US dairy herds, 68.1% are infected [4]. Financial losses due to the disease were estimated to be between $200 million and $250 million annually, in the US dairy industry alone [5]. Although still debated, the pathogen might play a role in pathogenesis of Crohn’s disease in humans [6,7].

Transmission of JD can occur mainly in three different ways. Calves can become infected in utero and via MAP-contaminated milk or colostrums [1,2]. The other transmission route, affecting all age classes, is fecal-oral, since the pathogen is also shed in the feces of infected individuals [8]. The disease causing pathogen can persist in the environment for more than one year [9]. Infectiousness of animals, for all modes of transmission, is thought to depend on the amount of bacteria shed into the environment [10]. However, it can vary from less than 2 to more than 10 years before clinical symptoms are visible [11]. During this period, shedding at different levels may occur [11]. Shedding levels have been categorized in low, medium and high shedders depending on the number of colonies obtained by fecal culture or Ct value obtained by quantitative polymerase chain reaction. For example, Whitlock et al. [12] used 10 and 50 colonies/tube to separate low, medium and high shedders. Smith et al. found an association of shedding levels (low and high) with milk production of the MAP-infected cattle [13].

A variety of tests for JD is available, all with advantages and disadvantages. Fecal culture is the most definitive; however, the test is costly and takes up to 16 weeks to perform [14]. Polymerase chain reaction (PCR) based methods are faster but are more expensive than fecal culture test [14]. Enzyme-linked immunosorbent assay (ELISA) tests measure specific antibodies in blood samples. These tests are easy to perform, results are available within a week and the cost is less than one third of the cost for fecal culture [14]. However, the sensitivity relative to fecal culture is low, especially for low-shedding animals [12]. In 2006, Eda et al. [15] reported that sensitivity of ELISA test for JD can be improved by using ethanol extract of MAP and named the new ELISA, ethanol vortex ELISA (EVELISA).

Several management strategies to control JD are recommended. To decrease transmission fecal-orally, strategies include manure management to prevent contamination of feed and water, tilling contaminated pastures and general hygiene [14]. Calves require extra attention because of their higher susceptibility [10,16] than adults. Strategies to prevent transmission via milk or colostrums include not pooling colostrums, only using colostrums from test-negative cows and feeding calves with pasteurized milk [14]. Culling test-positive animals is also recommended as a control strategy [14]. In that case, decisions to cull depend on the test used. Through modeling, we investigate the effects of various transmission routes on prevalence and economic outcomes of some testing regimes.

To analyze cost-effectiveness of ELISA-based JD control measures, an agent-based, discrete time model was developed to simulate JD dynamics in a dairy herd. The model incorporates contact structure, stochastic variation in demographic rates and disease dynamics. Two ELISAs with different sensitivities (i.e. current ELISA and EVELISA) were compared for their cost effectiveness. Our model includes some realistic features that were not included in two models [17,18] which considered the cost-effectiveness of testing regimes. Details of the comparison of these two models with our model will be given in the discussion section.

In the next section, we describe our model in detail. The third section gives our numerical results showing the prevalence changes over time and economic analysis. We finish with a discussion of our results.

## Materials and methods

### Model description

A discrete time, stochastic, agent-based model was created and implemented using NetLogo. We describe the model construction using the standardized overview, design concepts, and details (ODD) protocol [19].

#### Purpose

The model was created to simulate JD and population dynamics in a dairy herd. The model may be used to provide insight into the importance of various routes of transmission and to investigate the epidemiological effects and economic impact of different diagnostic tests and management strategies.

#### Entities, state variables, and scales

Agents are defined as cows and represent individual cows on a dairy farm. As shown in Table 1, cows have the integer state variables of age, days since a strong positive diagnostic test, and days since a weak positive diagnostic test. These three state variables are reported in terms of days. Cows also have several binary state variables that allow them to behave collectively in a number of different contexts. The values of different Boolean state variables may indicate age group, disease status, location, or pregnancy status.

**Table 1:** **List of state variables used in the agent-based model for JD**

**Category**	**Age group**	**Disease status**	**Location**	**Pregnancy**	**Diagnostic testing**
**State variables**	calf	healthy	Calf-hutch	Pregnant	Tested-strong
	heifer	exposed	Heifer-group		Tested-weak
	adult	high-shedder	Pasture		Days-after-strong
		low-shedder	Pregnancy-group		Days-after-weak
			Maternity-barn		Weak-positive
			Lactation-barn		

All state variables have the ability to change over time. State variables from the age group, location, and pregnancy categories only vary based on the progression of time. Changes in state variables from the disease status and diagnostic testing categories along with the addition and removal of cows include elements of stochasticity.

**Figure 1:**
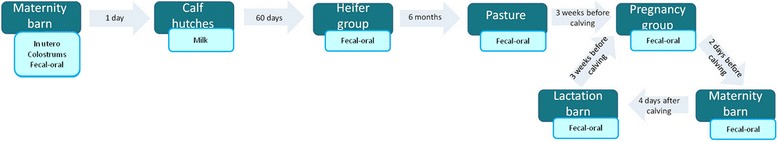
**The scheme by which individuals move through different spatial compartments.** Modes of disease transmission that occur in each compartment are indicated. Green squares indicate location of animals.

Each time step represents 1 day, and the model is run for up to 3650 days for population dynamics and for economic analysis. The model assumes random mixing of cows within a specific location which is a feature of each cow’s current status. As shown in Figure 1, the location includes calf hatches, heifer group pen, pasture, pregnancy group pen, maternity barn and lactation barn.

#### Process overview and scheduling

During each time step, cows execute a specific sequence of processes. The first process is “grow”, which includes aging and the possibility of giving birth. The second is “have-chance-of-infection”, the third is “progress-in-disease”, and the fourth is “survive”. The fifth is “move”, which also incorporates diagnostic testing practices. All cows execute one of the five processes (or steps) before the entire population moves on to the next process in the sequence. Cows complete each process in a randomized turn-taking order. Any updates made to a particular cow’s state variables as it is executing a process are implemented immediately. Selling and buying of heifers occur once a week and twice a year, respectively, to keep the population size consistent.

#### Design concepts

##### Basic principles

The underlying design of the model is based largely on the common dairy farm management practice of grouping cows by age and providing separate housing for each group. The features of these separate housing environments vary based on the particular needs or characteristics of the individuals in each group. Due to the age differences and spatial variations in the environment for each group, different housing environments are likely to have different risks for transmission and routes of infection. The separation into six spatial compartments allows for these differences to be incorporated into the model. The spatial compartments, routes of disease transmission, and scheme for time-based movement between compartments used in the model are represented in Figure 1.

The separate spatial compartments allow for different routes of disease transmission in different age groups and spatial areas to be varied and tested independently. This helps provide insight into which age-specific or area-specific management practices would be most effective.

The structure of the diagnostic testing and actions taken due to test results are based on common and recommended practices. Individuals may test as strong positive, weak positive, or negative for JD either by ELISA or EVELISA. Individuals identified as strong positives are removed from the herd, and individuals identified as weak positives are tagged as such and their colostrums are not used to feed calves. These actions do not take place until 7 days pass in order to account for time between testing and obtaining results.

##### Emergence

JD status of each animal is defined as susceptible (uninfected), exposed, low-shedding and high-shedding. In this study, the term, “exposed”, means that an animal is infected with MAP but not shedding MAP in its feces, milk and/or colostrums. The presence of each low shedding or high shedding individual contributes to the infection of other individuals in its respective spatial compartment via fecal-oral transmission, and thus also affects the change in prevalence indirectly. The dynamics of the prevalence values over time are expected to change when different disease transmission routes in different spatial compartments are turned on, turned off, or changed. They are also expected to change when certain management practices are applied to the entire herd, such as a testing and culling strategy.

##### Interaction

Results of interactions between individuals are assumed based on different routes of disease transmission. Individuals born into the herd interact directly with their mothers during “chance of in-utero” infection and when drinking colostrums. New calves may also directly drink colostrums from one other mother in the maternity barn. Individuals are assumed to interact indirectly through fecal-oral transmission. Low shedding and high shedding individuals contribute to the environment within their respective spatial compartments, and the environment is assumed to contribute to the probability of infection of uninfected individuals in that spatial compartment.

##### Collectives

Animals (agents) are grouped into collectives in three different ways: age class, state of disease, and spatial compartment. The age classes are calf, heifer, and adult. We assume that calves become heifers after weaning and that heifers become adults after giving birth for the first time. Animals in the calf group have ages 0–60 days, animals in the heifer group have ages 61–730 days, and animals in the adult group have ages 731 days and above. Age classes are important when creating initial age distributions. States of disease include exposed, low shedding, and high shedding. Different disease states influence the infection dynamics of the entire population. The assigned spatial compartment is based on age and modeled after common dairy management practices. Locations include the maternity barn, the calf hutches, heifer group housing, the pasture, pregnancy group housing, and the lactation barn. Each compartment has its own set of transmission rates based on agents present in the group.

##### Stochasticity

The processes of age initialization, disease initialization, successful female birth, natural mortality, disease transmission, disease progression, and diagnostic test results are all assumed to be stochastic. The stochasticity in age and disease initialization produces variability in the initial conditions. The purpose of the stochasticity in successful female birth, natural mortality, disease dynamics, and diagnostic test results is to replicate real-world frequencies of events.

##### Observation

The prevalence of the disease in each stage and the number of individuals in each age class are tracked at each time step. Running totals of number of cows sold, number of cows bought, number of diagnostic tests administered, and number of cows culled are kept.

#### Initialization

During the model setup, cows are created and randomly assigned ages and infection status within a predetermined age structure. Of the initial cows, 10% are designated as calves, 40% are designated as heifers, and 50% are designated as adults. Each calf is then assigned a random age between zero and sixty, each heifer a random age between 61 and 730, and each adult a random age between 731 and 2190. Each cow is then placed in the appropriate spatial compartment according to Table 2.

**Table 2:** **Days for translocation of animals**

**Spatial compartment**	**Calf hutches**	**Heifer group housing**	**Pasture**	**Pregnancy group housing**	**Maternity barn**	**Lactation barn**
Age	0–60	61–179	180–710	711–728, 1066–1124, 1462–1520, 1858–1916	729–735, 1125–1131, 1521–1527, 1917–1923	736–1065, 1132–1461, 1528–1859, 1923–2190

The numbers of each group are calculated based on the initial population, and members of each age group are randomly selected to change their infection status. Initial prevalence of JD used in this study is shown in Table 3.

**Table 3:** **Initial JD prevalence**

	**Exposed**	**Low-shedding**	**High-shedding**
Calves	35%	0%	0%
Heifers	31%	4%	0%
Adults	25%	8%	2%

#### Input data

The model does not use input data to represent time-varying processes.

#### Functions (submodels)

The Grow function advances each animal’s age by 1 day. Depending on the new age, the animal may move to a new location (heifer group pen, maternity barn etc. shown in Figure 1), new age category (i.e. heifer or adult), and/or give a birth. If an animal gives a birth, its offspring has a 50% of chance to be female and has a chance to be infected (in utero or through colostrums). The parameters used for these routes of infection are shown in Additional file 1. If the calf does not become exposed, it remains healthy. The new calf is then placed in the maternity barn.

The have-chance-of-infection function implements infection of healthy calves through colostrums from a second mother and fecal-oral transmission of all healthy cows.

If a calf is healthy newborn in the maternity barn and colostrums from another dam is set to be on, it has a 50% chance of drinking from any non-pregnant adult animal in the maternity barn. One dam is selected at random. If the dam selected is a low shedder and it has not been classified as a weak positive through ELISA or EVELISA testing, the calf has a chance of becoming exposed. Similarly, if the dam selected is a high shedder and has not been classified as a weak positive through ELISA or EVELISA testing, the calf has a higher chance of becoming exposed than the case the dam is a low shedder. Also, calves can become infected through drinking milk from low and high shedders. Fecal-oral transmission occurs independently in each compartment. This probability of fecal-oral transmission is defined by the following function:

$$ infection\  chanc{e}_{comp}=\beta \frac{\left(1-\gamma \right) low\  shedder{s}_{comp}+\left(\gamma \right) high\  shedder{s}_{comp}}{total\  populatio{n}_{comp}} $$

Where, the “*infection chance*_*comp*_” is the probability of an animal in the compartment getting infected in 1 day. The subscript “comp” indicates the compartment where the animal locates on that day. *low-shedders*_*comp*_*, high-shedder*_*comp*_ and *total-population*_*comp*_ are the number of animals in the compartment on that day. β is the transition rate (set to 0.002, 0.0002, and 0.00002 for calves, heifers and adults, respectively) from susceptible (uninfected) animals to exposed (infected but not shedding) animals. Parameter γ, which is set to 0.9 for the entire study, allows for high shedders to impact infection transmission more than low shedders. Fecal-oral route infections occur in all the compartments except for calf hatches where calves are well separated.

In the Progress-in-disease (transition from exposed to low shedder and then to high shedder) function, each low shedder has a probability of becoming a high shedder and each exposed cow has an exposed-to low chance of becoming a low shedder.

The Survive (natural mortality rates) function simulates removal from the farm due to natural death or timely removal. There are separate daily survival rates for calves in their first 48 h of life, other calves, heifers, and adults. The survival rate for the first 48 h includes unsuccessful births, and the adult survival rate includes removal due to old age or disease. Cows that are not successful in the survive function are removed from the population.

During the Move function, each cow may be relocated to the appropriate new compartment based on its new age assigned in Grow.

The Test function implements ELISA (or EVELISA) testing. The model allows for the use of ELISA testing, EVELISA testing, or neither test, with multiple testing up to four times a year. A cow may test as a strong positive, a weak positive, or a negative and are tagged as such. Details are described in our previous paper [17]. There are different probabilities for each test result depending on the fecal shedding status of the animal (i.e. no-shedding (susceptible or exposed), low shedding, or high shedding). Seven days (time required to get test results back) after the testing, cows that tested as strong positives are removed from the herd and those that tested as weak positives are tagged as known weak positives. Cows that are classified as known weak positives do not contribute colostrums toward feeding calves.

The Buy function occurs every buying-interval (182 days) when the total number of cows is less than the initial population. Cows are introduced until the total number of cows is equal to the initial population. New cows are springing heifers and are placed the pregnancy group housing. These new cows are initialized as healthy, exposed or low shedding according to the initial prevalence which was determined based on the regional prevalence.

The Sell function acts every 7 days if the total number of cows is greater than the initial population. Cows classified as heifers are removed from the population until the total number of cows is equal to the initial population.

### Simulations

Simulations were run to provide insight into the relative importance of each route of disease transmission, the effect of eliminating fecal-oral transmission in the pasture, and the epidemiological and economic effects of employing a test and cull strategy using the ELISA test or the EVELISA test. Due to the stochastic nature of the model, ten runs were simulated for each parameter combination. Results were measured at each time step in terms of prevalence of exposed cows, prevalence of low shedding cows, prevalence of high shedding cows, and total prevalence. For the simulations measuring economic impact, results were reported in terms of net gain or net loss at the end of each iteration.

Tested scenarios are as follows:

1. Contribution of each transmission pathway (no ELISA-based intervention).
  All transmission pathways possible
  No fecal-oral transmission
  No transmission through MAP contaminated milk
  No transmission through contaminated colostrums
  No in utero transmission
2. Impact of ELISA-based interventions (all transmission pathways possible)
  No testing
  ELISA test-based interventions (once or four times a year)
  EVELISA test-based interventions (once or four times a year).

### Economic analysis

The revenue was calculated to be (sales of milk and culled cows) minus (payments for replacement heifers and testing). Other costs are assumed to be consistent in each scenario and whereas not included in the calculation.

## Results

**Figure 2:**
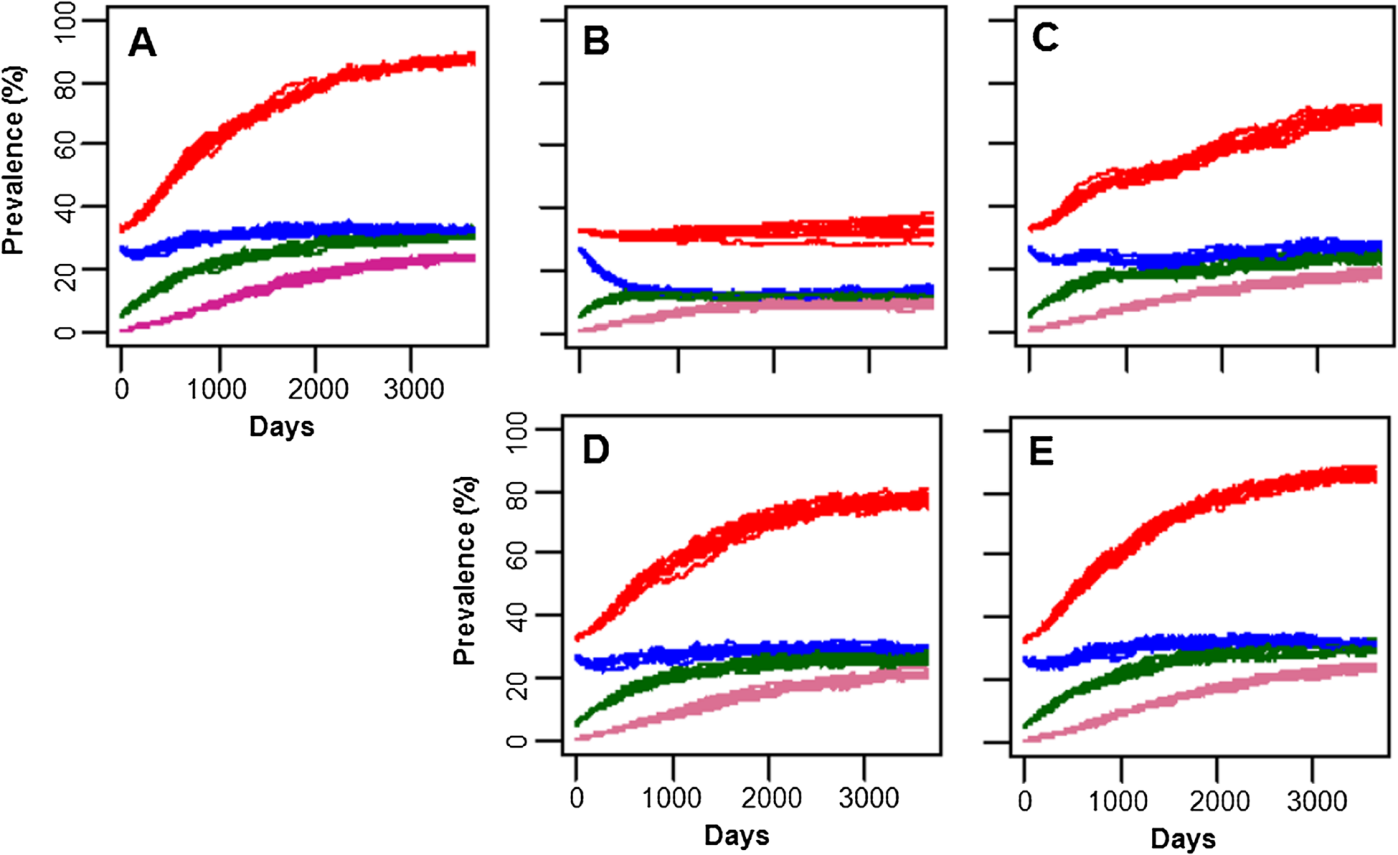
**Population dynamics of JD in a dairy farm simulated by the JD agent-based model.** **A**: All transmission; **B**: no fecal-oral; **C**: no milk transmission; **D**: no colostrums transmission; **E**: no vertical transmission. Red: Total infected animals; Blue: Exposed animals; Green: Low shedding animals; Purple: high shedding animals.

Population dynamics of JD in a dairy farm was simulated using the agent-based model developed in this study. When all the transmission routes were included, the total prevalence of JD (total infected animals, exposed + low shedders + high shedders) increased from the initial level (average ± standard deviation: 33.1 ± 0.2%), to 87.7 ± 1.7% in the 10 year simulation (Figure 2A). The prevalence of JD in each age group at the end of the 10-year simulation was 21.9 ± 5.4%, 32.9 ± 1.6% and 41.5 ± 2.6% for calves, heifer and adults, respectively. When fecal-oral route transmission was removed from the simulation, the total prevalence did not increase but persisted (Figure 2B). Removal of transmissions through milk (Figure 2C), colostrums (Figure 2D) and vertical transmission (Figure 2E) had much less effect on the increase in prevalence and the total prevalence at the end of the simulations were about 71.6 ± 1.8%, 77.3 ± 1.9% and 86.1 ± 1.6%, respectively.

**Figure 3:**
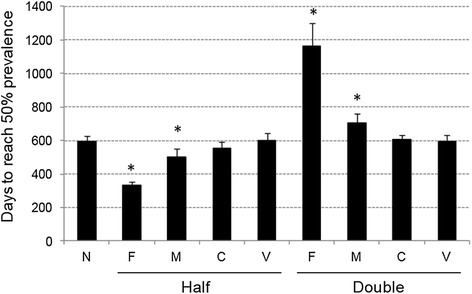
**Influence of assumed parameters on transmission of MAP.** In this study, assumed values were used for infection rates for fecal-oral (F), milk (M), C (colostrums) and V (vertical) transmissions. Simulations were run with halved or doubled each infection rate. Each bar represents days that required for the total prevalence (exposed + low shedding + high shedding animals) to reach 50%. The error bars indicate standard deviation of data obtained by 10 simulations. Statistical significance among the group was detected by ANOVA test. Asterisks indicate that a statistical significant between the data and the original data (N) was detected by pair-wise t- test with Bonferroni and Holm adjustments.

**Figure 4:**
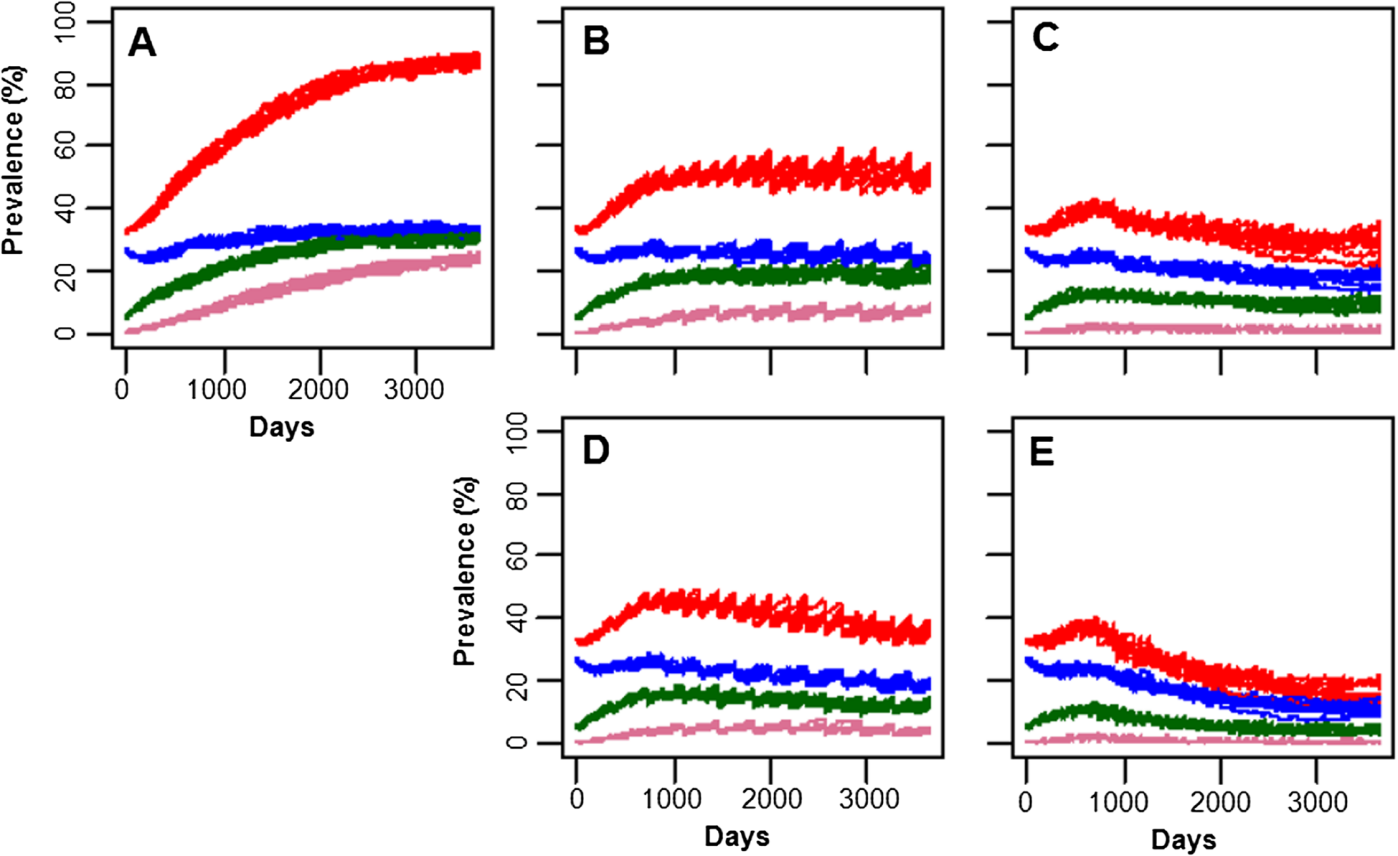
**Population dynamics of JD in a dairy farm simulated by the JD agent-based model.** **A**: No testing; **B**: ELISA (once/year); **C**: EVELISA (once/year); **D**: ELISA (4 times/year); **E**: EVELISA (4 times/year). Red: Total infected animals; Blue: Exposed animals; Green: Low shedding animals; Purple: high shedding animals.

In this study, assumed values were used for infection rate for each transmission route (i.e. fecal-oral, milk, colostrums and vertical) as listed in Additional file 1. To evaluate influence of each assumed parameter on prevalence, simulations were run with halved or doubled level of the parameter. Influence on prevalence was evaluated by finding number of days that required for the total prevalence to reach 50%. As shown in Figure 3, infection rate for fecal-oral transmission route, followed by that for milk transmission route, had the most significant influence on rate of prevalence increase. Changing infection rates for colostrums and vertical transmission routes did not result in any statistically significant difference. With all the transmission modes included, ELISA- or EVELISA-based control measures were applied in the model (Figure 4). When results of ELISA and EVELISA testing (once a year) was used to control JD in the dairy farm, the prevalence after 10 year simulation was reduced to 50.9 ± 1.6% and 36.2 ± 1.6%, respectively (Figures 4B and C). As shown in Figures 4D and E, more significant reductions (ELISA: 28.4 ± 3.5% and EVELISA: 15.7 ± 1.9%) was observed when the testing was conducted quarterly (four times a year).

Using recent values of milk, replacement heifer, culled cow and ELISA testing, revenues of the simulated dairy farm was calculated under different scenarios of JD control. With annual testing, ELISA- and EVELISA-based control measures reduced revenue of the simulated dairy farm and levels of the reduction were greater for EVELISA (Table 4). When testing frequency was increased to four times a year, ELISA- and EVELISA-based control showed higher revenues than no-testing scenario at the initial prevalence of 10 and 20%; however, the difference was not statistically significant. Statistically significant differences were observed for ELISA- and EVELISA-based controls when the simulations were run for 20 years. The increases in revenue are 1.79 and 2.06 million US dollars/20 years for ELISA and EVELISA, respectively.

**Table 4:** **Economic analysis of ELISA-based control measures**

**Testing**	**Initial prevalence (%)** ^**a**^	**Control**	**Average** ^**b**^	**SD**	**Difference**
Once	5	None	32.4	0.22	
		ELISA	32.0	0.08	−0.38^c^
		EVELISA	31.9	0.18	−0.44^c^
	10	None	31.9	0.14	
		ELISA	31.4	0.14	−0.53^c^
		EVELISA	31.3	0.19	−0.61^c^
	20	None	31.5	0.18	
		ELISA	31.0	0.18	−0.45^c^
		EVELISA	30.8	0.23	−0.64^c^
Four times	5	None	32.4	0.22	
		ELISA	32.2	0.18	−0.13
		EVELISA	32.2	0.17	−0.18
	10	None	31.9	0.14	
		ELISA	32.0	0.16	0.01
		EVELISA	32.0	0.11	0.07
	20	None	31.5	0.18	
		ELISA	31.6	0.14	0.09
		EVELISA	31.6	0.18	0.17

^a^Prevalence of shedders in adult population. The unit of the monetary numbers is million dollars/10 years ^a^. Ten percent is the same distribution of prevalence shown in Table 3; ^b^Average revenue calculated from results of 10 simulations; and ^c^statistically different (ANOVA followed by t test with Bonferroni and Holm adjustments, *p* < 0.01) from no ELISA-based control.

## Discussion

Epidemiological studies of MAP have been hampered by the fact that currently-used diagnostic tests are incapable of detecting the early (latent) stage of MAP infections. For better understanding of JD epidemiology, mathematical modeling approach has been employed since the early ‘90s. Collins et al. [20] presented the first mathematical model describing the behavior of JD in an open herd. For evaluation of JD management strategies, Groenendaal et al. [21] developed stochastic models—named “JohneSSim”. Since 2008, Mitchell et al. [22] and Lu et al. [23,24] developed mathematical models that incorporated a “transient shedding” within the calf population. Lu et al. [24] employed a stochastic compartmental model to better evaluate fadeout of JD in dairy herds. These works are reviewed in a recent publication [25].

In these previous models, there were some missing factors that would be important for understanding of JD epidemiology. For example, most of these models assumed that animals become resistant to MAP infection after one year of age; however, some findings [16,26,27] indicated that adult animals could also get infected with MAP and developed JD. Also, the contact structure in a dairy herd was not incorporated in mathematical models of JD with exception of a recent report [28]. Further, only a limited number of studies employed agent-based modeling approach. We therefore elected to develop an agent-based model incorporating MAP infection in adult animal population and contact structure. Agent-based model captures emergent phenomena, provides a natural description of the modeled system and is flexible especially in geospatial models.

Our model predicted that, if no control measure was applied, the initial prevalence of JD in the modeled herd 33.1 ± 0.2%) would increased to 87.7 ± 1.7% after a 10 year-simulation, which is similar to the prediction obtained by a previous modeling work [18]. The end-point prevalence of 90% may sound very high but is possible because the prevalence include exposed (latent) animals whose number was reported in this special issue to be 2.5 times higher than that of fecal culture positive animals [29] and the prevalence of fecal culture positive animals could reach close to 40% [30].

Similar to the model presented in this study, our previous model [17] was developed using a contact structure in a dairy herd but was based on a set of difference equations. The previous model predicted that use of EVELISA was more cost effective (40 US dollars/cow/10 years) than the current ELISA test. The most closely related work to this study was conducted by Kudahl et al. in 2007 [18]. Their model is based on SimHerd which is an agent-based model but, in contrast to our study, contact structure was not considered. Another major difference is that our study used sensitivities of commercial ELISA and EVELISA obtained by testing a same set of filed samples whereas the previous study used assumed values for the ELISA with a higher sensitivity (improved ELISA). Their model predicted that, after 10 years of ELISA-based control, the improved ELISA is more cost-effective (70–80 Euro/cow/10 years) than current ELISA when initial prevalence was set to 25% and test-&-cull control strategy (quarterly for < 4 years old animals and annually for older animals) was implemented in their model. In our study, EVELISA was predicted to be more cost-effective (67.5US dollars/cow/10 years) than the current ELISA when initial fecal culture positive prevalence was set to 10% and quarterly test-&-cull was implemented for 20 years in the model.

Currently, ELISA testing for JD control is conducted only once a year [31]. Our model predicted that even though the increase in JD prevalence could be slowed down by applying annual ELISA-based control, there would be a negative impact on revenue. Although the quarterly test-&-cull control was able to significantly reduce the prevalence and also predicted to be cost-effective, it will increase labor for testing and is currently not realistic. Recent work has demonstrated that an on-site diagnostic device for JD could be developed by using a capacitance sensing approach [32], and once fully developed, such a device would make it easier and cheaper to implement a quarterly test and cull procedure.

## Additional file

Additional file 1:
**List of parameters used in this study.** Parameter values and their source were listed [4,12-14,17,18,23,30,33,34].

## Abbreviations

JD: Johne’s disease
MAP: *Mycobacterium avium* subsp. *paratuberculosis*
ELISA: Enzyme-linked immunosorbent assay
EVELISA: Ethanol-vortex ELISA
PCR: Polymerase chain reaction
ODD: Overview, design concepts, and details
