# Supplementary material for: Agent-based model for Johne’s disease dynamics in a dairy herd
Source: Vet Res. 2015 Jun 19;46(1):68. doi: 10.1186/s13567-015-0195-y (PMC4474466; doi:10.1186/s13567-015-0195-y)
Supplement: Additional file 1: — List of parameters used in this study. Parameter values and their source were listed [4,12-14,17,18,23,30,33,34]. [file 13567_2015_195_MOESM1_ESM.docx]

**Additional file 1 List of parameters used in this study.**

| **Description** | **Value in model** | **Source** |
| --- | --- | --- |
| Initial ratio of calves to heifers to adults | 0.1:0.4:0.5 | Expert’s opinion |
| Last day cow is considered calf (before transition to heifer) | 60 days | Expert’s opinion |
| First day cow is considered a heifer | 61 days | [4] |
| Last day cow is considered heifer (before transition into adult) | 730 days | Expert’s opinion^a^ |
| First day cow is considered an adult and first calving date | 731 days | [4] |
| Maximum age (number of days) at which cow can be initialized | 2190 days | Expert’s opinion |
| First day calf is in maternity barn (day calf is born) | 0 days | Logic |
| Last day calf is in maternity barn | 1 day | Expert’s opinion |
| First day calf is in calf hutch | 2 days | Expert’s opinion |
| Last day calf is in calf hutch | 60 days | Expert’s opinion |
| First day heifer is in heifer group housing | 61 days | Expert’s opinion |
| Last day heifer is in heifer group housing | 179 days | Expert’s opinion |
| First day heifer is on pasture | 180 days | Expert’s opinion |
| Last day heifer is on pasture | 710 days | Expert’s opinion |
| First day in pregnancy group housing | 711 + n*calving interval days  (*n*=0,1,2,3…) | Expert’s opinion |
| Last day in pregnancy group housing | 728 + n*calving interval days  (*n*=0,1,2,3…) | Expert’s opinion |
| First day in maternity barn | 729 + n*calving interval days  (*n*=0,1,2,3…) | Expert’s opinion |
| Last day in maternity barn | 735 + n*calving interval days  (*n*=0,1,2,3…) | Expert’s opinion |
| First day in lactation barn | 736 + n*calving interval days  (*n*=0,1,2,3…) | Expert’s opinion |
| First day dry period | 1066 + n*calving interval days  (*n*=0,1,2,3…) | [4] |
| Chance of calf being female | 0.5 | Logic |
| Number of days between calvings | 396 days | [4] |
| Daily chance of survival for first 2 days | 0.9273618495 | [4] |
| Daily chance of survival for calves | 0.9997775314 | [4] |
| Daily chance of survival for heifers | 0.9999502369 | [4] |
| Daily chance of survival for adults | 0.99906211 | [4] |
| Time between selling events | 7 days | Expert’s opinion |
| Time between buying events | 182 days | Expert’s opinion |
| Time between testing events | 365 days | Expert’s opinion |
| Initial prevalence of exposed calves | 0.35 | [12,30] |
| Initial prevalence of exposed heifers | 0.31 | [12,30] |
| Initial prevalence of exposed adults | 0.25 | [12,30] |
| Initial prevalence of low-shedding heifers | 0.04 | [12,30] |
| Initial prevalence of low-shedding adults | 0.08 | [12,30] |
| Initial prevalence of high-shedding adults | 0.02 | [12,30] |
| Disease progression rate from exposed to low-shedding (chance per day) | 0.0014010507 | [23] |
| Disease progression rate from low-shedding to high-shedding (chance per day) | 0.000781617 | [12,23] |
| Chances of calf getting infected inutero from a low-shedding mother | 0 | [18] |
| Chances of calf getting infected inutero from a high-shedding mother | .22 | [18] |
| Chances of calf getting infected from drinking colostrum from a low-shedding adult | .16 | [18] |
| Chances of calf getting infected from drinking colostrum from a high-shedding adult | .36 | [18] |
| Chance of getting infected fecal-orally from the environment per area | 0.9999724652 * ((1 - HS-coef) * number of low-shedders in area + (HS-coef) * number of high-shedders in area)/number of cows in area. | Assumed |
| Coefficient indicating how many times a high-shedder contributes to the chance of getting infected fecal-orally compared to a low-shedder | 0.9 | Assumed |
| chance of infection when each cow in compartment is infected | 0.002 | Assumed |
| weighs the chance of heifers being infected | 0.1 | Assumed |
| weights the chance of adults being infected | 0.01 | Assumed |
| coefficient indicating how many times a calf has the chance of getting infected via milk | 0.7 | Assumed |
| Chance of calf becoming infected when there is an infected mother in the calf-hutch | 0.0001 | Assumed |
| Number of days from test to results | 7 days | Expert’s opinion |
| chance that a non-shedder will have a positive ELISA test | 0.0 | [17] |
| chance that a non-shedder will have a strong positive ELISA test | 0.0 | [17] |
| chance that a low-shedder will have a positive ELISA test | 0.53 | [17] |
| chance that a low-shedder will have a strong positive ELISA test | 0.15 | [17] |
| Chance that a high-shedder will have a positive ELISA test | 0.89 | [17] |
| chance that a high-shedder will have a strong positive ELISA test | 0.65 | [17] |
| chance that a non-shedder will have a positive EVELISA test | 0.04 | [17] |
| chance that a non-shedder will have a strong positive EVELISA test | 0.0 | [17] |
| chance that a low-shedder will have a positive EVELISA test | 0.9 | [17] |
| chance that a low-shedder will have a strong positive EVELISA test | 0.36 | [17] |
| chance that a high-shedder will have a positive EVELISA test | 1.0 | [17] |
| chance that a high-shedder will have a strong positive EVELISA test | 0.81 | [17] |
| milk production in lbs per cow per day | 74.38 lbs | [4] |
| value of milk per 100 lb | $12.88 | [33] |
| amount less than milk-prod that a high shedder produces | .218  (modified) | [13] |
| Amount less than milk-prod that a low shedder produces | .019  (modified) | [13] |
| cost per elisa and Evelisa test | $5.00 | [14] |
| cost to buy one springing heifer | $1,800.00 | Expert’s opinion |
| Revenue by selling one healthy cow | $1730 | [4] |
| Average weight (lbs) of a dressed cull cow (meat only) | 622 lbs | [4] |
| Average worth of 1lb cull cow meat | $1.873 | [34] |
| The cost for the entire operation | .1903 | [34] |
